# Cerebral blood flow and white matter alterations in adults with phenylketonuria

**DOI:** 10.1016/j.nicl.2023.103550

**Published:** 2023-12-09

**Authors:** Leonie Steiner, Raphaela Muri, Dilmini Wijesinghe, Kay Jann, Stephanie Maissen-Abgottspon, Piotr Radojewski, Katarzyna Pospieszny, Roland Kreis, Claus Kiefer, Michel Hochuli, Roman Trepp, Regula Everts

**Affiliations:** a Department of Diabetes, Endocrinology, Nutritional Medicine and Metabolism, Inselspital, Bern University Hospital and University of Bern, Switzerland; b Division of Neuropaediatrics, Development and Rehabilitation, Department of Paediatrics, Inselspital, Bern University Hospital and University of Bern, Switzerland; c Support Center for Advanced Neuroimaging (SCAN), University Institute of Diagnostic and Interventional Neuroradiology, Inselspital, Bern University Hospital and University of Bern, Switzerland; d Translational Imaging Center (TIC), Swiss Institute for Translational and Entrepreneurial Medicine, Bern, Switzerland; e Laboratory of Functional MRI Technology (LOFT), Stevens Neuroimaging and Informatics Institute, Keck School of Medicine, University of Southern California, USA; f Magnetic Resonance Methodology, Institute of Diagnostic and Interventional Neuroradiology, Inselspital, Bern University Hospital and University of Bern, Switzerland

**Keywords:** Phenylketonuria, Arterial spin labeling, White matter, Cerebral blood flow, Cognition, Spectroscopy

## Abstract

**Background:**

Phenylketonuria (PKU) represents a congenital metabolic defect that disrupts the process of converting phenylalanine (Phe) into tyrosine. Earlier investigations have revealed diminished cognitive performance and changes in brain structure and function (including the presence of white matter lesions) among individuals affected by PKU. However, there exists limited understanding regarding cerebral blood flow (CBF) and its potential associations with cognition, white matter lesions, and metabolic parameters in patients with PKU, which we therefore aimed to investigate in this study.

**Method:**

Arterial spin labeling perfusion MRI was performed to measure CBF in 30 adults with early-treated classical PKU (median age 35.5 years) and 59 healthy controls (median age 30.0 years). For all participants, brain Phe levels were measured with ^1^H spectroscopy, and white matter lesions were rated by two neuroradiologists on T2 weighted images. White matter integrity was examined with diffusion tensor imaging (DTI). For patients only, concurrent plasma Phe levels were assessed after an overnight fasting period. Furthermore, past Phe levels were collected to estimate historical metabolic control. On the day of the MRI, each participant underwent a cognitive assessment measuring IQ and performance in executive functions, attention, and processing speed.

**Results:**

No significant group difference was observed in global CBF between patients and controls (*F* (1, 87) = 3.81, *p* = 0.054). Investigating CBF on the level of cerebral arterial territories, reduced CBF was observed in the left middle and posterior cerebral artery (MCA and PCA), with the most prominent reduction of CBF in the anterior subdivision of the MCA (*F* (1, 87) = 6.15, *p* = 0.015, surviving FDR correction). White matter lesions in patients were associated with cerebral blood flow reduction in the affected structure. Particularly, patients with lesions in the occipital lobe showed significant CBF reductions in the left PCA (*U* = 352, *p* = 0.013, surviving FDR correction). Additionally, axial diffusivity measured with DTI was positively associated with CBF in the ACA and PCA (surviving FDR correction). Cerebral blood flow did not correlate with cognitive performance or metabolic parameters.

**Conclusion:**

The relationship between cerebral blood flow and white matter indicates a complex interplay between vascular health and white matter alterations in patients with PKU. It highlights the importance of considering a multifactorial model when investigating the impact of PKU on the brain.

## Highlights

• Global cerebral blood flow (CBF) did not differ between patients with PKU and controls.
• CBF was reduced in the middle and posterior cerebral artery in patients with PKU.
• White matter lesions and axial diffusivity were associated with reductions of CBF.
• Vascular health and white matter alterations interact in patients with PKU.

## 1 Introduction

**Fig. 1:**
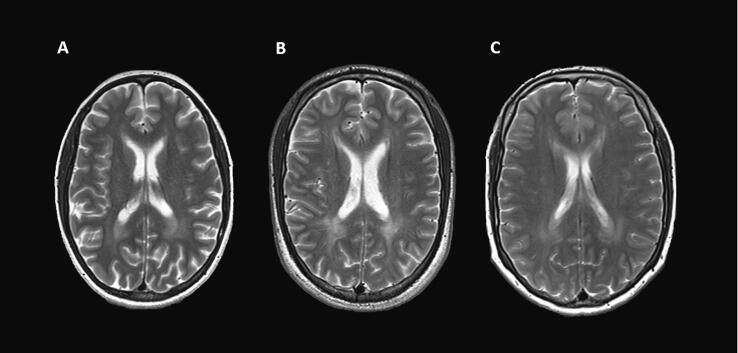
Typical periventricular white matter lesions in adults with PKU. *Note.* Axial slices of T2-weighted images showing typical white matter hyperintensities in three adult patients with PKU (A-C). These abnormalities are most frequently seen in the periventricular white matter of the brain and appear as increased signal (hyperintensities) on T2-weighted images. All patients gave their consent to publish their MR image.

Phenylketonuria (PKU) results from a defective function of the enzyme phenylalanine hydroxylase (Blau et al., 2010). The impaired conversion of the amino acid phenylalanine (Phe) into tyrosine leads to high Phe levels and low-to-normal plasma tyrosine concentrations. If infants and children with PKU are left untreated, severe and irreversible neurological and cognitive sequelae occur, such as mental disabilities, behavioral and psychiatric problems, tremor, or epilepsy (Blau et al., 2010, Luna et al., 2023). An early-initiated dietary protein restriction combined with Phe-free amino acid supplementation prevents the development of severe clinical symptoms (van Spronsen et al., 2021). However, mild difficulties in cognitive and psychosocial functioning often remain in adulthood despite early and continuous treatment (van Spronsen et al., 2021).Fig. 1.

The etiology of patients’ cognitive and psychosocial difficulties is likely multifactorial (Blau et al., 2010). Previous studies found structural and functional brain alterations, which may underlie the remaining functional difficulties. White matter alterations are the most consistent finding reported in the literature (Anderson and Leuzzi, 2010, Antenor-Dorsey et al., 2013, González et al., 2018, Hawks et al., 2019, Hood et al., 2015, Mastrangelo et al., 2015, Muri et al., 2023, Peng et al., 2014, Thompson et al., 1990, White et al., 2010). Particularly, periventricular white matter hyperintensities are typically found in patients with PKU combined with an increase of frequency and severity from the second decade of life onwards (Mastrangelo et al., 2015). Further, these white matter changes have been associated with metabolic parameters, such as concurrent blood and brain Phe levels (Vermathen et al., 2007) and cognitive outcomes (Antenor-Dorsey et al., 2013). An age-dependent individual susceptibility to Phe has been proposed for the white matter signal alteration (Mastrangelo et al., 2015). Besides white matter alterations, gray matter characteristics such as thickness, volume, and density are also affected, predominately in posterior areas of the brain (Aldridge et al., 2020, Bodner et al., 2012, Christ et al., 2016, Muri et al., 2022, Pérez-Dueñas et al., 2006, Pfaendner et al., 2005). One study has shown moderate correlations between gray matter thickness and working memory performance (Muri et al., 2022). Complementary to structural alterations, decreased functional connectivity within the default mode network (DMN) (Christ et al., 2012), decreased neural activation in the DMN during an inhibitory task (Sundermann et al., 2020) and altered neural activation during a working memory fMRI task (Abgottspon et al., 2022) have been shown in some studies with early-treated patients with PKU, whereas others did not present altered neural activation in executive brain networks (Sundermann et al., 2011). In the study by Abgottspon et al. (2022), neural activation in the working memory network was related to concurrent Phe, tyrosine, and tryptophan levels. Christ et al. (2012) found a similar relationship between high recent Phe levels and decreased functional connectivity.

Apart from the described structural and functional brain alterations in patients with early-treated PKU, altered brain vascularization and cerebral perfusion might further contribute to the multifactorial alterations in this disease. Since cerebral blood flow (CBF) is generally coupled to glucose metabolism and neuronal activity (Grade et al., 2015, Hoge and Pike, 2001), it is a surrogate marker of brain function. Thus, the assessment of CBF could provide additional information about functional brain mechanisms and its contributing impact on the clinical symptoms in patients with early-treated PKU. On the one hand, white matter hyperintensities have been associated with reduced CBF in the affected regions (Crane et al., 2015). On the other hand, a positron emission tomography (PET) study in patients with PKU found no global alterations in cerebral blood flow (CBF) (Hasselbalch et al., 1996). However, focusing solely on global CBF one may overlook regional blood flow variations. Assessing CBF in different vessel territories provides a more comprehensive understanding of regional differences and helps identify pathological processes. Considering both global and regional CBF measures enhances our understanding of cerebral perfusion. Additionally, PET indirectly measures CBF and requires invasive radioactive tracers, which is a disadvantage.

CBF in different vessel territories can reliably and non-invasively be measured with arterial spin labeling (ASL) perfusion MRI. ASL is of particular interest owing to its quantitative nature and capability to determine CBF in different cortical cerebral arterial territories without the need for invasive tracers (Grade et al., 2015). ASL generates an image by magnetically “labeling” water molecules as endogenous tracers and selective radiofrequency irradiation inverts the magnetization of the arterial blood water in the region to which it is applied. A downstream measurement is taken as labeled spins exchange into the tissue of interest (Grade et al., 2015).

According to our knowledge, no studies have examined cerebral blood flow using ASL in patients with early-treated PKU. Hence, we aimed to compare cerebral blood flow between patients and controls in different cortical cerebral arterial territories, including the anterior cerebral artery (ACA), middle cerebral artery (MCA), and posterior cerebral artery (PCA) in both hemispheres separately. Further, we assessed the relationship between CBF, white matter, metabolic parameters, and cognitive performance.

## 2 Materials and methods

### 2.1 Participants

Data for this cross-sectional study were derived from the PICO study (Phenylalanine and its Impact on Cognition), a clinical trial investigating the impact of Phe on cognitive functions, mood, and depression in a longitudinal randomized control trial ((Trepp et al., 2020); clinicaltrials.gov NCT03788343). The present study focuses on the baseline data collected in the framework of the PICO study. Thirty early-treated patients with classical PKU and 59 healthy demographically comparable controls were recruited. The patient and control sample included in this study encompassed all or a subset of participants included in two prior studies investigating cross-sectional structural and functional imaging data (Muri et al., 2022 (patients: *n* = 30, controls: *n* = 54) and Abgottspon et al., 2022 (patients: *n* = 20, controls: *n* = 40), respectively). Patients (13 females, median age = 35.5 years, IQR = 12.3, age range = 19–48 years) were recruited through the University Hospitals of Bern, Zurich, Lausanne, Basel, and the Cantonal Hospital St. Gallen (Switzerland), the University Hospitals in Ulm and Hamburg (Germany), and Innsbruck (Austria). Inclusion criteria were (1) ≥ 18 years, (2) positive newborn screening test for PKU, and (3) treated with a Phe-restricted diet within the first 30 days of life. Exclusion criteria were (1) Phe concentrations exceeding 1600 μmol/l six months before study participation, (2) untreated vitamin B12 deficiency, (3) pregnancy, (4) breastfeeding, (5) unwillingness to follow a highly efficient contraception during study participation, (6) history of neurological disorders, (7) severe psychiatric conditions, or (8) any contraindication for MRI (e.g., metal implants or epilepsy).

Control participants (33 males, 26 females, median age = 30.0 years, IQR = 11.0, age range = 18–53 years) were included if they were 18 years or older and were recruited in Bern and Zurich through advertisements and word-of-mouth. Exclusion criteria for controls were (1) a history of neurological disorders, (2) severe psychiatric conditions, and (3) any contraindication for MRI (e.g., metal implants or epilepsy).

Education was assessed in all patients and controls with the categories defined as follows: 1—Secondary Education; 2—Apprenticeship; 3—Vocational Education; 4—High School; 5—College of Higher Education; 6—Bachelor or equivalent; 7—Master or equivalent; 8—Doctorate. These categories were then grouped into three educational groups: (1) High school (category 1), (2) College/Job training (categories 2–6), and (3) Graduate school (categories 7–8).

Written informed consent was obtained from all participants before study participation. The study was performed according to the Declaration of Helsinki and was approved by the Cantonal Ethics Committee Bern, Switzerland (2018–01609).

### 2.2 Clinical data

#### 2.2.1 Metabolic parameters

Plasma Phe concentrations were determined based on blood sampling, performed after an 8–12 h overnight fasting period and before the MRI examination. Plasma amino acid profiles were assessed using a Biochrom 30 amino acid analyzer (Saturn & Venus models). This analysis was performed through high-performance ion-exchange liquid chromatography, coupled with post-column photometric detection of amino acids derivatized with ninhydrin. Further details about laboratory analysis methods can be found elsewhere (Abgottspon et al., 2022). Twenty-two out of 30 patients were above the recommended level of 600 µmol/l proposed by the current European guidelines (van Wegberg et al., 2017) with a median Phe level of 741 µmol/l for the whole patient sample (IQR = 358, min value = 380, max value = 1208). Historical metabolic control was determined with the index of dietary control (IDC), and both plasma and dry blood spots were included. This index is calculated as the mean of the yearly medians of all available Phe levels measured throughout patients’ lives. Four age categories were created according to distinct developmental periods: 0–5 years, 6–12 years, 13–17 years, and 18 + years. Each patient had to have a minimum of 10 measurements per age category to calculate the IDC (Weglage et al., 1996). Further, brain Phe levels were measured using ^1^H magnetic resonance spectroscopy as described under section 2.3 Neuroimaging.

#### 2.2.2 Cognitive assessment

The cognitive assessment was performed after the MRI assessment. General intelligence was assessed using the subtests Matrix Reasoning, Vocabulary, Arithmetic, and Symbol Search from the Wechsler Adult Intelligence Scale Fourth Edition (WAIS-IV) (van Ool et al., 2018). Executive functions were assessed based on the model of Miyake et al. (2000) and included working memory (n-back task of the Test of Attentional Performance (TAP) (Zimmermann & Fimm, 2009)), inhibition, and cognitive flexibility (Color-Word Interference Test conditions 3 and 4; Delis-Kaplan Executive Function System (D-KEFS) (Delis et al., 2001)). Attention was measured using the TAP subtests Alertness, Divided Attention, and Sustained Attention (Zimmermann & Fimm, 2009). Processing speed was assessed with the subtest Symbol Search of the WAIS-IV (Petermann, 2012). The cognitive tasks are described in more detail in the published PICO study protocol (Trepp et al., 2020).

### 2.3 Neuroimaging

MRI and ^1^H MR spectroscopy data were acquired on a 3 T Siemens Prisma MRI scanner at the Translational Imaging Center (TIC) within the Swiss Institute for Translational and Entrepreneurial Medicine (SITEM), Bern, Switzerland. The scanner had a 64-channel head coil with an integrated mirror allowing participants to watch a nature documentary during structural image acquisition. The MRI exam was performed after an 8–12 h overnight fasting period.

#### 2.3.1 MRI acquisition

A T1-weighted image (MPRAGE) was collected for all participants (TR = 1950 ms, TE = 2.26 ms, TI = 900 ms, TA = 4:34, flip angle = 9, in-plane resolution = 1 × 1 mm, slice thickness = 1 mm, number of slices = 176, FOV = 256 mm × 256 mm, matrix = 256 × 256). For lesion load analyses, T2-weighted images were obtained (axial: 0.5 × 0.5 × 3.0 mm, TR = 4800 ms, TE = 88 ms, TA = 1:04 min; sagittal: 1.0 × 1.0 × 4.0 mm, TR = 3000 ms, TE = 84 ms, TA = 0:26 min; coronal: 1.0 × 1.0 × 4.0 mm, TR = 3000 ms, TE = 84 ms, TA = 0:23 min). Diffusion-weighted images were obtained using a spin-echo EPI sequence, encompassing 122 non-collinear directions, with a preceding b = 0 reference volume. The acquisition parameters were as follows: TR = 3700 ms, TE = 87 ms, TA = 7:55, slice thickness = 2.2 mm (isotropic), number of slices = 56, phase encoding direction = anterior-posterior, acceleration factor = 2, *q*-space weightings = 3, q-space max. *b*-value = 3000 s/mm2, and full *q*-space coverage.

To assess CBF in the gray matter, we adopted a pulsed arterial spin labeling (pASL) sequence with a FAIR (slice-selective and nonslice-selective inversion pulse) labeling scheme, which was performed at rest (Kim, 1995). The following parameters were used: acquisition time, TA = 4.59 min, repetition time, TR = 8000 ms, echo time, TE = 16.18 ms, bolus duration = 800 ms, inversion time, TI = 1500 ms, FOV = 192mmx192mm, flip angle = 180°, voxel size = 1.5 x 1.5 x 3.0 mm, 4 label/control pairs. Additionally, an M0 image (TA = 2.16 min) for quantifying CBF was recorded with TR = 8000 ms and TI = 7000 ms.

#### 2.3.2 Acquisition of ^1^H MR spectroscopy

The acquisition of ^1^H MR spectroscopy is described in more detail in a previous publication of our study group (Muri et al., 2023). A semi-LASER sequence with the manufacturer’s second-order shimming routine was used to acquire MR spectra. A volume of interest of 50 × 75 × 20 mm^3^ (or reduced to 50 × 65 × 20 mm^3^ depending on head geometry) was semiautomatically placed in supraventricular white and gray matter with a small preponderance of white matter (∼5mm spacing to the roof of the lateral ventricles) (Hoefemann et al., 2019). TE was set to 35 ms and TR to 2500 ms. The transmit frequency was set to 7.3 ppm for water-suppressed spectra, and 256 acquisitions were averaged from two batches of 128 scans (12 min total scan time). For eddy current and phase correction, unsuppressed reference scans of the water signal were recorded for each subject. For water referencing and evaluation of cerebrospinal fluid (CSF) signal contributions, an additional series of eight scans with different TEs (35, 50, 75, 100, 140, 200, 400, 1000 ms) was recorded with a TR of 6000 ms.

#### 2.3.3 Analysis of arterial spin labeling

ASL data were preprocessed and quantified using MATLAB (Version R2019b; The MathWorks Inc., Natick, MA, USA), SPM12 and FSL (FMRIB’s Software Library). ASL data preprocessing included output of some quality control metrics including framewise displacement, temporal SNR and DVARS of the label and control images, as well as temporal SNR and spatial standard deviation of the resulting CBF images. These metrics allow for detection of datasets with low quality. After ASL time series were realigned to correct for motion artifacts, pair-wise subtraction of label and control images was performed to generate perfusion images (Kim, 1995). Brain extraction was done using FSL-bet, and quantitative CBF maps were generated using an in-house MATLAB toolbox (LOFT-CBF). LOFT-CBF toolbox is freely available including open-source code in GitHub repository: www.github.com/kayjann/LOFT-CBF. The toolbox uses a single-compartment kinetic model and performs voxel-wise calibration using the M0 image. Individual mean-CBF maps were calculated by averaging across CBF time-series maps. The resulting CBF maps were then co-registered with the T1w MRI and normalized to the Montreal Neurological Institute (MNI) template space. Regional CBF values for different cortical perfusion territories (ACA, MCA, PCA) were extracted based on the Tatu perfusion territory atlas (Tatu et al., 1998). The quantified perfusion is indirectly related to the quantification of CBF in units of milliliters of blood per 100 g of tissue per minute (Grade et al., 2015).

#### 2.3.4 White matter lesion load

White matter lesions of patients and controls were rated by two board-certified neuroradiologists (PR & KP) on T2-weighted images. Raters were blinded to the medical history, age, and sex of the subjects. White matter abnormalities were defined as high signal on T2-weighted images, which is assumed to reflect intramyelinic edema in early-treated patients with PKU (Reddy et al., 2018). White matter lesions were rated on a scale from 0 − 12, according to the suggestions by Pietz et al. (1996). Frontal, parietal, temporal, and occipital lobes were rated on a 3-point scale. 0 point was given if no white matter was affected, 1 point if deep white matter was affected, and 2 points if images showed subcortical white matter involvement. Additionally, brainstem and cerebellum were each rated on a 2-point scale, with 0 points for no involvement and 2 points indicating white matter involvement. For more details, see our previous publication (Muri et al., 2023). Although many semiautomated and automated methods are available to assess white matter hyperintensities, their reproducibility and comparability still lacks in-depth characterization (Caligiuri et al., 2015). Deep learning-based algorithms are in development (Balakrishnan et al., 2021), but still require validation. Thus, well validated visual scores remain useful.

#### 2.3.5 DTI analysis

We preprocessed DTI data using FSL software (version 6.0.5) following the pipeline of Maximov et al. (2019) including noise corrections, Gibbs ringing corrections, eddy-current and motion corrections (FSL eddy), skull-stripping (FSL-BET), bias field corrections using ANTs, and spatial smoothing (FSL fslmaths). We applied DTIFIT for diffusion tensor modeling.

Voxel-Wise Analysis (TBSS): For voxel-wise analysis, we utilized tract-based spatial statistics (TBSS) to assess group differences in DTI metrics. The method projected fractional anisotropy (FA), mean diffusivity (MD), axial diffusivity (AD), and radial diffusivity (RD) onto a white matter skeleton generated from FA maps. Group differences were statistically evaluated using “randomise” with 5000 permutations, and Threshold-Free Cluster Enhancement was used for multiple comparison correction, with further *p*-value adjustment for multiple DTI metrics using the Bonferroni method.

Region-of-Interest Analysis (ROI Analysis): In the ROI analysis, we focused on 12 white matter tracts extracted from the JHU ICBM-DTI-81 atlas (Mori et al., 2008, Oishi et al., 2008) and the XTRACT HCP Probabilistic Tract atlas (Warrington et al., 2020), known for differences in pediatric and mixed-age patient samples with PKU. These tracts included the genu, body, and splenium of the corpus callosum, optic radiation, anterior, superior, and posterior corona radiata, inferior longitudinal fasciculus, superior longitudinal fasciculus, anterior and posterior limbs of the internal capsule, and the external capsule. Only DTI metrics showing significant differences in the voxel-wise analysis were included in the ROI analysis.

#### 2.3.6 ^1^H MR spectroscopy analysis

Data processing was performed in JMRUI (Stefan et al., 2009), which included frequency alignment, eddy current correction, signal averaging, and residual water signal removal with a Hankel Lanczos singular value decomposition filter. The spectra were scaled by the size of the parenchymal water signal from a biexponential fit of the TE-series (thus compensating for CSF water signal contributions). Phe was quantified as described by Kreis et al. (2009). Spectra were fitted in FiTAID (Chong et al., 2011) for the upfield (20 metabolites and a macromolecular background spectrum) and the downfield part. The fitting model for the largely ill-defined downfield part of the spectrum was based on summed spectra from healthy subjects and patients with PKU. Besides the simulated responses of Phe, homocarnosine (hCs), and N-acetyl aspartate (NAA), 20 Voigt lines (with heuristically optimized frequencies as well as Lorentzian and Gaussian broadening) were included. The Phe content was quantified based on the assumed water content of the parenchymal signal estimated from literature recommendations (Near et al., 2021) and white and gray matter content assumptions for the region of interest. Lacking published values for 3 T, T_1_ and T_2_ relaxation effects were considered assuming relaxation constants of 890 ms and 124 ms for T_1_ and T_2_, respectively, comparable to mean values of the methylene signal of creatine for white and gray matter (Träber et al., 2004). Quality control of MR spectra was implemented both, at the acquisition (control of adequate shim before recording of water suppressed data, visual inspection of incoming single shot data for changes of water suppression efficacy and frequency drifts) and at the post-processing stage (visual control of fit residuals for appearance of unusual features).

### 2.4 Statistical analysis

All analyses were performed using the statistical software SPSS version 25.0. Data visualization was conducted with R version 4.1.2. All variables were tested for normality with the Shapiro–Wilk test. Mean values of cognitive variables were compared between groups using one-sided non-parametric Quades’ ANCOVAS with age as a covariate. Global gray matter and regional cortical perfusion territory CBF values were compared between groups using two-sided non-parametric Quades’ ANCOVAS with age and sex as covariates. CBF analyses were corrected for age and sex due to evidence for sex and age differences in CBF metrics (Burley et al., 2021, Mokhber et al., 2021). To investigate the effect of lesion load, we adopted non-parametric Quades’ ANCOVA with an independent variable with three groups (patients with lesions/patients with no lesions/ healthy controls) and age and sex as covariates. To investigate the relationship between CBF, white matter integrity, metabolic parameters, and cognition, we applied partial correlations (Spearman), with age and sex as covariates. False discovery rate (FDR) correction was employed for all analyses. Uncorrected *p*-values are presented in the manuscript, and it is stated in brackets whether *p*-values survive FDR correction. Post hoc tests were performed using Mann– Whitney U tests. Rank-biserial correlation coefficients (*r_rb_*) were calculated as effect sizes with the corresponding 95 % confidence interval CI. Effect sizes were interpreted as suggested by Funder and Ozer (2019): very small *r* ≥ 0.05, small *r* ≥ 0.10, medium *r* ≥ 0.20, large *r* ≥ 0.30, and very large *r* ≥ 0.40.

### 2.5 Data availability

Upon reasonable request and with the consent of the study team, the study data are available from the corresponding author.

## 3 Results

### 3.1 Demographic and clinical data

Patients (*n* = 30) and controls (*n* = 59) did not differ with respect to demographic variables such as age, sex, and education level (Table 1). Historical Phe concentrations were not available for all patients. In the age band 0 and 5 years, the IDC of one patient (out of 17 patients, 5.9 %) was above the recommended level of 360 μmol/l (range = 129–401 μmol/l). In the age band 6 and 12 years, the IDC of five patients (out of 19 patients, 26.3 %) was above 360 μmol/l (range = 153–431 μmol/l). During adolescence, six patients (out of 21 patients, 28.6 %) were above the recommended level of 600 μmol/l (range = 135–783 μmol/l). In adulthood, 11 out of 18 patients (61.1 %) were above 600 μmol/l (range = 408–1494 μmol/l).

**Table 1:** Demographic and metabolic data.

		Patients (n = 30) Median (IQR)	Controls (n = 59) Median (IQR)	*χ2* / *U*	*p*
Baseline data					
	Age, years	34.50 ± 14.25	30.00 ± 11.00	801.5	0.47
	Sex, females, *n* (%)	13 (43.3 %)	26 (44.1 %)	0.00	0.95
	Education, *n* (%)			5.63	0.56
	High school	1 (3.3 %)	4 (6.8 %)		
	College/job training	24 (80.0 %)	48 (81.3 %)		
	Graduate school (master/doctorate)	4 (13.3 %)	7 (11.9 %)		
Metabolic data					
	Brain Phe (mmol/l)	0.14 ± 0.04	0.04 ± 0.01	0.00	<0.01
	Plasma Phe (µmol/l)	741 ± 375.5	–	–	–
	Plasma tyrosine (µmol/l)	38 ± 12.0	–	–	–
	Plasma tryptophan (µmol/l)	37.5 ± 10.5	–	–	–
	IDC 0–5 years (µmol/l) (*n* = 17)	276 ± 130.5	–	–	–
	IDC 6–12 years (µmol/l) (*n* = 19)	320 ± 134.0	–	–	–
	IDC 13–17 years (µmol/l) (*n* = 21)	459 ± 284.5	–	–	–
	IDC ≥ 18 years (µmol/l) (*n* = 18)	640 ± 570.0	–	–	–

*Note.* IQR = interquartile range. *p* <.05 (two-sided); *χ2* = Chi-square test; *U* = Mann-Whitney *U* test; IDC = Index of Dietary Control. The numbers in brackets next to the IDC variables indicate the number of patients for whom the IDC could be calculated in the specific age category.

Regarding current Phe levels (same day as MRI assessments), 22 out of 30 individuals with PKU (73.3 %) had concurrent plasma Phe concentrations above the suggested target range (120–600 µmol/l). Concurrent tyrosine concentrations were toward the lower end of the reference range, with 15 patients (56.7 %) having concentrations below the reference range of 40–100 µmol/l. In four patients (13.3 %), tryptophan concentrations were below the reference range of 30–90 µmol/l. Brain Phe tissue content ranged between 0.10 and 0.29 mmol/l for patients with a median of 0.14 mmol/l, whereas for controls they ranged between 0.02 and 0.06 mmol/l with a median of 0.04 mmol/l. Group differences in brain Phe levels were statistically significant (*p* <0.001). There was a significant positive correlation between brain and plasma Phe levels (*r* = 0.819, *p* <0.001).

Regarding cognition, group medians of all cognitive domains were within the normal range in the patient sample (see T-scores and scaled scores in Table 2). Nevertheless, patients with PKU had a significantly lower IQ and performed significantly worse than controls in the subdomains processing speed, working memory, cognitive flexibility, and sustained attention (Table 2). All significant group differences survived FDR correction. Results of the cognitive outcomes of the present sample were published previously with a slightly smaller control sample (Muri et al., 2022). During recruitment phone calls, the patients were asked if they had any comorbidities. Three patients responded to have comorbidities: obesity, asthma, and one patient with generalized muscle pain that improved significantly after taking duloxetine.

**Table 2:** Cognitive performance in patients and controls.

			Patients (n = 30)Median (IQR)	Controls (n = 59)Median (IQR)	U	p	r_rb_ [95 % CI] for r_rb_
IQ	Intelligence	Index scores^1^	97.0 (16.0)	110.0 (±20.0)	520	<0.01*	−0.39 [−0.58, −0.15]
	Processing Speed	Scale scores (time in seconds)^1^	10.0 (3.5)	12.0 (±11.0)	497.5	<0.01*	−0.82 [−0.88, −0.74]
Executive functions	Working memory	Raw scores (accuracy in %)^1^	94.5 (7.0)	98.0 (±3.0)	527	<0.01*	−0.41 [−0.60, −0.17]
	Inhibition	Scale scores (time in seconds)^1^	10.0 (2.5)	11.0 (±4.0)	673	0.11	−0.19 [−0.39, 0.03]
	Cognitive flexibility	Scale scores (time in seconds)^1^	10.0 (2.0)	11.5 (±3.0)	502	<0.01*	−0.31 [−0.50, −0.10]
Attention	Alertness	T-scores (median reaction time in milliseconds)^1^	45.5 (9.8)	47.0 (±12.0)	735	0.22	−0.13 [−0.33, 0.09]
	Divided attention	T-scores (total omissions)^1^	48.0 (13.0)	53.0 (±13.0)	725	0.30	−0.11 [−0.32, 0.11]
	Sustained attention	T-scores (sd of reaction time in milliseconds)^1^	46.0 (9.0)	52.0 (±10.5)	513	<0.01*	−0.31 [−0.60, −0.10]

*Note.* IQR = interquartile range; *U* = Mann-Whitney *U*-statistic; *p* = *p*-value; effect sizes are reported as rank-biserial correlations (rrb) for Mann-Whitney *U*-tests; CI = confidence interval; sd = standard deviation, ^1^the higher/the more positive the score, the better performance, * survived FDR-correction.

### 3.2 Cerebral blood flow

#### 3.2.1 Global cerebral blood flow

Group analyses of global CBF (non-parametric Quades’ ANCOVA with age and sex as covariates) showed a non-significant group difference between patients and controls (*F* (1, 87) = 3.81, *p* = 0.054, effect size *r_rb_* = 0.19 [CI −0.03, 0.38]), with patients showing lower CBF than controls.

#### 3.2.2 Analysis in different cortical arterial perfusion territories (ACA, MCA, PCA)

Detailed results of the Quades’ ANCOVAs for different cortical arterial perfusion territories (ACA, MCA, PCA) are displayed in Table 3. Reduced CBF was observed in the left MCA and PCA in patients compared to controls (left MCA, *F* (1, 87) = 4.81, *p* = 0.03; left PCA *F* (1, 87) = 4.80, *p* = 0.03), which however did not survive FDR correction. CBF in the ACA did not differ between groups.

**Table 3:** Cerebral blood flow (residuals) in patients with PKU and controls (all ROIs).

	Between-group differences in CBF											
	Left Hemisphere						Right Hemisphere					
CBF in different ROIs	PatientsMedian (IQR)	ControlsMedian (IQR)	*F*	*P*	*r_rb_*	[95 % CI] for r_rb_	PatientsMedian (IQR)	Controls Median (IQR)	*F*	*p*	*r_rb_*	[95 % CI] for r_rb_
ACA total	−0.03 (1.65)	0.03 (1.07	0.52	0.47	0.10	[−0.11, −0.31]	0.01 (1.11)	−0.03 (1.23)	0.10	0.76	0.01	[−0.18, −0.25]
ACA anterior	−0.06 (1.89)	−0.05 (0.85)	0.28	0.60	0.07	[−0.15, −0.28]	0.08 (1.71)	−0.10 (0.90)	0.24	0.63	0.01	−0.20, −0.23]
ACA posterior	−0.05 (1.61)	0.08 (1.13)	0.34	0.56	0.07	[−0.14, −0.28]	0.17 (1.34)	−0.07 (1.16)	0.32	0.75	0.04	[−0.28, −0.14]
MCA total	−0.33 (1.64)	0.17 (1.22)	4.81	0.03	0.22	[0.01, −0.42]	−0.24 (1.36)	0.01 (1.24)	3.42	0.07	0.19	[−0.04, −0.37]
MCA anterior	−0.43 (1.65)	0.18 (1.28)	6.15	0.015*	0.22	[0.04, −0.41]	−0.14 (1.34)	0.06 (1.16)	3.29	0.07	0.18	[−0.07, −0.35]
MCA middle	−0.36 (1.52)	0.08 (1.05)	4.20	0.04	0.22	[0.04, −0.42]	−0.32 (1.31)	0.13 (1.45)	3.26	0.08	0.18	[−0.04, −0.37]
MCA posterior	−0.39 (1.09)	0.09 (1.18)	3.14	0.08	0.20	[−0.02, −0.39]	−0.39 (1.25)	0.20 (1.50)	4.68	0.03	0.27	[0.06, −0.46]
PCA total	−0.33 (1.37)	0.14 (1.39)	4.80	0.03	0.23	[0.01, −0.42]	−0.36 (1.48)	0.10 (1.15)	2.33	0.13	0.20	[−0.07, −0.35]

*Note*: IQR = interquartile range. *p <.05 (two-sided); *F* = Quads’ ANCOVA. CBF is displayed as standardized residuals after regressing out the effect of age and sex on CBF raw values. CI, confidence interval, *p*-values are uncorrected. Results reaching significance after correction for multiple testing (FDR correction) are marked with *.

Analyses of different subdivisions of the MCA (including anterior, middle, and posterior MCA) revealed that CBF was significantly reduced in patients in the left anterior part of the MCA (*F* (1, 87) = 6.15, *p* = 0.015), which survived FDR correction. Further, CBF in the left MCA middle, left PCA as well as the right MCA posterior (left MCA middle: *F* (1, 87) = 4.20, *p* = 0.04; left PCA: *F* (1, 87) = 4.80, *p* = 0.03; right MCA posterior: *F* (1, 87) = 4.68, *p* = 0.03) was lower in patients, which did not survive FDR correction. CBF in subdivisions of the ACA did not differ between groups.

### 3.3 White matter and cerebral blood flow

Conspicuous white matter lesions were found in 29 out of 30 patients (96.7 %) and 2 out of 59 controls (3.4 %). In most patients, white matter lesions were bilateral, relatively symmetrical, periventricular, and diffuse as typically seen in patients with early treated PKU. None of the patients had white matter alterations due to leukodystrophy or small vessel disease. In patients, total white matter (WM) lesion scores ranged from 0 to 7 (median = 2.5, IQR = 1.75). The parietal lobe was most frequently affected (29/30, 96.7 %), followed by the occipital lobe (19/30, 63.3 %), frontal lobe (15/30, 50.0 %), temporal lobe (4/30, 13.3 %), brain stem (3/30, 10.0 %), and cerebellum (2/30, 6.7 %). WM lesion scores of > 1 were only found in the occipital lobe. Specifically, 5 out of 19 patients had a WM lesion score = 2 in the occipital lobe (n = 14: WM lesion score = 1), emphasizing that the occipital lobe is the most prominently affected structure in terms of white matter hyperintensity.

Based on the results presented above, we analyzed the WM lesion score in the occipital lobe (19/30 patients affected) and in the frontal lobe (15/30 patients affected) with respect to the CBF territories (ACA/MCA/PCA). As 29 out of 30 patients had conspicuous white matter lesions in the parietal lobe, an ANCOVA could not be run for this lobe. To investigate the effect of lesion load on CBF in the ACA, MCA and PCA, we adopted two separate non-parametric Quades’ ANCOVAs with age and sex as covariates and lesion load scores as independent variable. WM lesion load groups included (1) patients with lesions in the occipital/frontal lobe (WM lesion score ≥ 1 or higher), (2) patients without occipital lesions (WM lesions score = 0), and (3) healthy controls.

Analyses of the occipital lesion load score revealed a significant group effect of CBF in the left PCA (*F* (2, 86) = 3.2, *p* = 0.045). Post hoc analyses revealed that patients with lesions in the occipital lobe showed significantly lower CBF than controls in the left PCA (*U* = 352, *p* = 0.013, effect size *r_rb_* = 0.29 [CI 0.06, 0.49], surviving FDR correction). On the other hand, patients without any lesions showed no significant difference in CBF compared to controls in the left PCA (*U* = 75, *p* = 0.22, effect size *r_rb_* = 0.08 [CI −0.17, 0.31]). Post hoc analyses investigating patients with lesions in the occipital lobe versus patients without lesions in the occipital lobe revealed non-significant effects between the two patient groups with, however, moderate effect size (*U* = 282, *p* = 0.533, effect size *r_rb_* = 0.24 [CI −0.15, 0.55]), indicating lower CBF in patients with occipital lesions. Global CBF and CBF in other cerebral arteries did not differ based on the lesion load scores in the occipital nor frontal lobe (patients with vs. without lesions). Total lesion load score was not correlated with global CBF nor CBF in different territories.

**Fig. 2:**
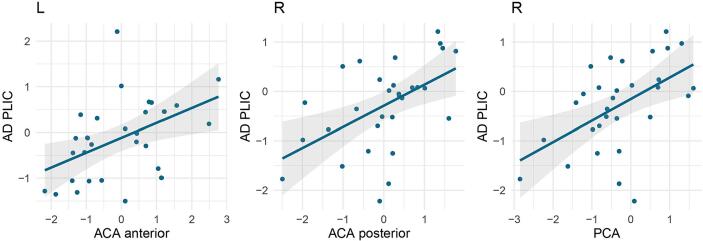
Positive correlations between axial diffusivity in the posterior limb of the internal capsule and cerebral blood flow in the ACA and PCA in the left and right hemisphere. Note. AD, Axial diffusivity; PLIC, posterior limb of the internal capsule; ACA, anterior cerebral artery; PCA, posterior cerebral artery. Correlations survived FDR correction.

Additionally, we investigated if white matter metrics measured with DTI are correlated with CBF metrics in patients with PKU. The results on the differences in DTI metrics between patients and controls are reported elsewhere (Muri et al., 2023). There are several correlations between white matter metrics and CBF with medium effect sizes (Supplementary Table S1). In particular, axial diffusivity in the posterior limb of the internal capsule was significantly correlated with CBF in the left ACA anterior (*r* = 0.51, *p* = 0.006), right ACA posterior (*r* = 0.51, *p* = 0.005) and right PCA (*r* = 0.53, *p* = 0.003, all these correlations survived FDR correction; see Fig. 2).

### 3.4 Relationship between CBF, metabolic control, and cognition

No statistically significant correlation was found between metabolic control (concurrent plasma Phe and brain Phe) and CBF after correcting for multiple testing (FDR correction) (see Table S2). However, there was a negative correlation with effect sizes of 0.68 and 0.71 between CBF and historic Phe levels in the age band 0–5 years and in the age band 6–12 years in the right MCA anterior (Table S2). Tyr and Trp were negatively associated with CBF (see Table S2), a relationship that did not survive FDR correction. Global and regional CBF did not correlate with cognitive performance in neither the patient nor the control sample (Table S2). Further, there is no significant relationship between metabolic control, lesion load and cognition in patients with early-treated PKU (Table S4).

## 4 Discussion

This study investigated CBF, white matter abnormalities, metabolic parameters, and cognition in adults with early-treated classical PKU compared to healthy controls. Patients presented CBF reductions in the MCA and PCA compared to controls, with the most significant reduction of blood flow in the left anterior MCA. White matter lesions in patients were associated with CBF reductions in the affected structures. Particularly, patients with lesions in the occipital lobe showed pronounced CBF reductions in the left PCA. Axial diffusivity in the posterior limb of the internal capsule was positively correlated with CBF in the ACA and PCA. Global and regional CBF did not correlate with cognitive performance or metabolic control (i.e., concurrent plasma Phe-levels, tyrosine, and tryptophan).

### 4.1 Group differences in cerebral blood flow

Adults with early-treated PKU displayed significantly lower CBF in the left MCA and PCA compared to controls, whereas CBF in the ACA did not differ between groups. Analyses of different subdivisions within the MCA (including anterior, middle, and posterior MCA) showed that patients’ CBF was particularly reduced in the left anterior and middle MCA, as well as in the right posterior MCA, compared to controls. The most prominent reduction in CBF, which survived FDR correction, was found in the left anterior MCA. This is in line with the previously published task-based fMRI study that includes two-thirds of the patients of the present study (Abgottspon et al., 2022). This study showed altered neural activation during a working memory task in the left middle frontal and superior frontal gyri, which are brain regions that are supplied by the anterior MCA. The reduction in CBF observed in the left MCA in patients with PKU might be attributed to several factors. The left anterior MCA and the associated frontal gyri may have a higher sensitivity to the metabolic disruptions caused by PKU, resulting in reduced CBF and altered brain function in these areas. Further, maturational effects might play a role in determining the susceptibility of the left vs. right hemisphere, as studies showed that the hemispheres mature differently and that there is a greater vulnerability of blood supply of the left than the right hemisphere (Njiokiktjien, 2006). This is observed as well in diseases such as unilateral stroke or unilateral epilepsy, which occur more frequently in the left than the right hemisphere (Njiokiktjien, 2006). Overall, the CBF of the left hemisphere seems more affected by PKU than the CBF within the right hemisphere in this study. A lateralized pattern of results was presented in a previous working memory fMRI study, where a hypoactivation of left brain areas and a hyperactivation of right brain areas is described in patients with PKU (Christ et al., 2013). Pronounced CBF reduction in the left hemisphere might as well be due to the enhanced left-sided structural changes in the gray matter metrics in this same group of patients, a finding that was also published previously (Muri et al., 2022). In line with this assumption, Crane et al. (2015) showed with voxel-based morphometric analyses that gray matter alterations are indeed associated with CBF alterations.

### 4.2 Association between CBF, white matter, and metabolic control

Patients with white matter lesions displayed pronounced CBF reductions in the affected region. Notably, patients with higher white matter lesion loads in the occipital lobe showed reduced CBF in the PCA. On the other hand, patients without occipital lesions showed no significant difference in CBF compared to controls. White matter hyperintensities are assumed to be caused by chronic hypoperfusion, blood–brain barrier breakdown, dysfunction of oligodendrocyte precursor cells, or venous collagenosis (Kim et al., 2020). A recent systematic review and meta-analysis showed that CBF is lower in subjects with more white matter hyperintensities (Shi et al., 2016). Further, the present study revealed that lower axial diffusivity in the internal capsule was significantly correlated with lower CBF in the ACA anterior, ACA posterior, and PCA. The association between CBF and white matter alterations has been demonstrated in individuals with white matter hyperintensities (Crane et al., 2015, Jann et al., 2021). Chen et al. (2013) presented in their study that there is a significant association between cortical CBF and white matter integrity throughout the brain. Interestingly, these associations persisted even after accounting for age, suggesting that the link between blood supply and white matter health is not solely attributed to aging. The study suggests that maintaining adequate blood supply to the brain is crucial for preserving white matter health among adults, and the decline in CBF with age may contribute to the deterioration of brain connective anatomy.

Worth noting might be that lower cerebral blood flow in the left anterior MCA was associated with elevated historical Phe levels during childhood. The large effect sizes (rrb = 0.68 and 0.71) did, however, not survive FDR correction. The direction of these negative associations between historical Phe and CBF aligns with prior research reporting a similar relationship between Phe levels and decreased functional connectivity (Christ et al., 2012). Additionally, a recent study within a subsample of the present cohort found an association between concurrent Phe levels and neural activation in the working memory network (Abgottspon et al., 2022). On a structural level, concurrent and historical Phe levels have been shown to be associated with gray matter (Bodner et al., 2012, Christ et al., 2016) and white matter integrity (Aldridge et al., 2020, Bodner et al., 2012, Christ et al., 2016, Pérez-Dueñas et al., 2006, Pfaendner et al., 2005).

Historical Phe levels indicated that patients following a strict diet were usually well-adjusted during childhood and adolescence, with increasingly higher Phe levels during adulthood. Regarding concurrent plasma Phe levels (same day as MRI assessment), only eight out of 30 patients (26.7 %) had plasma Phe concentrations within the target range suggested by the current European guidelines (van Wegberg et al., 2017). These results are reflected by the current literature, which shows that dietary adherence decreases after adolescence (Cazzorla et al., 2018).

Regarding cognitive functioning, patients’ IQ was within the normal range but significantly lower than the control group such as presented in previous studies (Feldmann et al., 2019, Jahja et al., 2017, Palermo et al., 2017). Also, processing speed, working memory performance, cognitive flexibility, and sustained attention were significantly reduced in patients compared to healthy controls, which is in accordance with previous studies (Ashe et al., 2019, Christ et al., 2010, Hofman et al., 2018, Luna et al., 2023). None of the cognitive domains were associated with metabolic parameters, global or regional CBF, or with white matter lesions. However, there was a significant correlation between white matter metrics measured with DTI and cognitive functions in the same patient sample, which was published before (Muri et al., 2023). This demonstrates that advanced imaging methods capable of assessing white matter microstructure, such as DTI, better detect potential associations between cognition functions and white matter abnormalities in patients with PKU. The non-significant association between the visually inspected white matter lesion scores and cognitive functions found in the present study might be due to the low variance in the lesion load variable, as almost all patients had a lesion score of 1 or 2, and lesions were present in multiple brain areas for the majority of patients. Nevertheless, visual inspection of white matter alterations is a valuable method as it can be performed in clinical routine.

Previous studies show that increases in cerebral perfusion may contribute to beneficial effects on cognitive functioning, as observed following increased physical activity (Joris et al., 2018) or cognitive training in healthy older adults (Mozolic et al., 2010). Cognitive function was shown to be more strongly affected by changes in cerebral metabolism than by changes in CBF during exercise (Ogoh, 2017). Therefore, it remains unclear how an alteration in CBF affects cognitive function in both patients with PKU and healthy adults.

### 4.3 Limitations

This study is subject to some limitations. First, the sample size is relatively small. This is, however, often an issue when conducting studies in the field of rare diseases such as PKU. The prevalence is only 1 per 8,000 newborns in Europe (Loeber, 2007). Normative modeling could be a useful approach as it can increase statistical power, impute or predict missing values, identify deviations from the norm, and aid in personalized medicine approaches for rare diseases. Secondly, we adopted pulsed arterial spin labeling (pASL) and refrained from Pseudo-Continuous Arterial Spin Labeling (pCASL) in this study, which allows for shorter imaging times and lower specific absorption rates (SAR). However, inherent limitations of pASL such as lower labeling efficiency, increased sensitivity to transit delays, and potentially less consistent whole-brain coverage compared to pCASL may have impacted the precision and comparability of our cerebral blood flow measurements. These are reasons why pCASL is the ASL technique currently recommended for clinical use (Lindner et al., 2023). Third, white matter lesions were rated for both hemispheres together, and a more detailed rating or even lesion quantification would be even more informative as far as the exact localization of the associations between white matter lesions and CBF is concerned. Fourth, to reduce heterogeneity only early-treated individuals with classical PKU following a Phe-restricted diet were included. It remains to be established whether the CBF characteristics examined are similar in individuals with mild PKU.

### 4.4 Conclusion

The present study revealed reduced CBF in the MCA and PCA in early-treated adults with PKU. Lower CBF was associated with white matter lesions as well as white matter integrity. The relationship between CBF alterations and white matter abnormalities underscores the complex interplay between vascular health and white matter pathology in patients with PKU. Overall, the findings suggest that subtle changes in functional brain metrics are intertwined with more pronounced structural alterations in adult patients with PKU. These results highlight the importance of adopting a multifactorial approach to studying the effects of PKU on the adult brain.

## Funding

The study was funded by two project grants of the 10.13039/501100001711Swiss National Science Foundation (192706 to R.E.; 175984 to R.K.) and a doc.CH grant awarded to R.M. (184453). Further financial contributions came from the 10.13039/501100008494Vontobel Foundation (Switzerland), the Bangerter Rhyner Foundation (Switzerland), a young investigator grant from the 10.13039/100018234Inselspital Bern (CTU grant) (Switzerland), the 10.13039/100022755Nutricia Metabolics Research Fund (The Netherlands) and the Fondation Rolf Gaillard pour la recherche en endocrinologie, diabétologie et métabolisme (Switzerland).

## CRediT authorship contribution statement

**Leonie Steiner:** Formal analysis, Validation, Visualization, Writing – original draft, Writing – review & editing. **Raphaela Muri:** Conceptualization, Data curation, Funding acquisition, Investigation, Project administration, Supervision, Writing – review & editing. **Dilmini Wijesinghe:** Formal analysis, Software, Validation. **Kay Jann:** Formal analysis, Software, Validation, Writing – review & editing. **Stephanie Maissen-Abgottspon:** Project administration, Data curation, Investigation. **Piotr Radojewski:** Formal analysis, Methodology. **Katarzyna Pospieszny:** Formal analysis, Methodology. **Roland Kreis:** Conceptualization, Methodology, Formal analysis. **Claus Kiefer:** Methodology. **Michel Hochuli:** Conceptualization, Supervision, Writing – review & editing. **Roman Trepp:** Conceptualization, Data curation, Funding acquisition, Investigation, Supervision, Resources, Writing – review & editing. **Regula Everts:** Conceptualization, Data curation, Funding acquisition, Investigation, Supervision, Resources, Writing – review & editing.

## Declaration of competing interest

The authors declare that they have no known competing financial interests or personal relationships that could have appeared to influence the work reported in this paper.

## Data Availability

Data will be made available on request.
