# Supplementary material for: Cerebral blood flow and white matter alterations in adults with phenylketonuria
Source: Neuroimage Clin. 2023 Dec 9;41:103550. doi: 10.1016/j.nicl.2023.103550 (PMC10716784; doi:10.1016/j.nicl.2023.103550)
Supplement: Supplementary data 1 [file mmc1.docx]

**Supplementary material**

Table S1. Relationship between CBF and white matter metrics measured with DTI in patients with early-treated PKU

| **Left Hemisphere** | Left AD PLIC  r | p value | Left AD SCR  r | p value | Left FA PLIC  r | p value | Left FA SCR  r | p value |
| --- | --- | --- | --- | --- | --- | --- | --- | --- |
| ACA anterior | **0.508*** | 0.006* | 0.342 | 0.075 | 0.426 | 0.024 | 0.321 | 0.095 |
| ACA posterior | 0.374 | 0.050 | 0.141 | 0.475 | 0.445 | 0.018 | 0.431 | 0.022 |
| MCA anterior | 0.438 | 0.020 | 0.468 | 0.012 | 0.380 | 0.046 | 0.367 | 0.055 |
| MCA middle | 0.308 | 0.111 | 0.183 | 0.352 | 0.414 | 0.029 | 0.424 | 0.024 |
| MCA posterior | 0.328 | 0.089 | 0.124 | 0.528 | 0.464 | 0.013 | 0.249 | 0.202 |
| PCA | 0.396 | 0.037 | 0.317 | 0.100 | 0.381 | 0.046 | 0.379 | 0.047 |
| **Right Hemisphere** | Right AD PLIC |  | Right AD SCR |  | Right FA PLIC |  | Right FA SCR |  |
| ACA anterior | 0.264 | 0.174 | 0.235 | 0.228 | 0.177 | 0.367 | 0.162 | 0.411 |
| ACA posterior | **0.512*** | 0.005* | 0.178 | 0.364 | 0.241 | 0.216 | 0.172 | 0.381 |
| MCA anterior | 0.241 | 0.217 | 0.233 | 0.232 | 0.073 | 0.710 | 0.161 | 0.413 |
| MCA middle | 0.355 | 0.064 | 0.304 | 0.116 | 0.239 | 0.220 | 0.336 | 0.081 |
| MCA posterior | 0.182 | 0.355 | 0.001 | 0.995 | 0.357 | 0.062 | 0.248 | 0.204 |
| PCA | **0.539*** | 0.003* | 0.235 | 0.228 | 0.219 | 0.262 | 0.255 | 0.190 |

*Note.* r, correlation coefficient; AD, axial diffusivity; PLIC, posterior internal capsule, L, left; R, right; SCR, superior corona radiate; ACA, anterior cerebral artery; MCA, middle cerebral artery, PCA, posterior cerebral artery; *correlation surviving correction for multiple comparisons (FDR correction).

**Table S2.** Relationship between CBF and metabolic control in patients with early-treated PKU (correlation coefficients).

| Metabolic parameters | Global CBF | Left MCA | Left MCA anter. | Left  MCA  middle | Left MCA post. | Left PCA | Right MCA | Right  MCA  anterior | Right  MCA middle | Right MCA posterior | Right PCA |
| --- | --- | --- | --- | --- | --- | --- | --- | --- | --- | --- | --- |
| Brain Phe (mmol/l) | -0.06 | 0.12 | -0.18 | 0.18 | 0.10 | -0.09 | -0.05 | -0.21 | -0.02 | 0.06 | 0.11 |
| Plasma Phe (µmol/l) | -0.17 | -0.04 | -0.29 | 0.04 | -0.10 | -0.04 | -0.21 | -0.39 | -0.14 | -0.11 | -0.20 |
| Tyrosine (µmol/l) | -0.37 | -0.41 | -0.39 | -0.39 | -0.38 | -0.34 | -0.36 | -0.42 | -0.32 | -0.15 | -0.17 |
| Tryptophan (µmol/l) | -0.43 | -0.42 | -0.31 | -0.47 | -0.32 | -0.35 | -0.47 | -0.50 | -0.38 | -0.32 | -0.20 |
| IDC 0-5 years | -0.29 | -0.27 | -0.46 | -0.12 | -0.49 | -0.14 | -0.35 | -0.68 | -0.38 | -0.33 | -0.28 |
| IDC 6-12 years | -0.41 | -0.33 | -0.44 | -0.22 | -0.58 | -0.24 | -0.44 | -0.71 | -0.48 | -0.41 | -0.29 |
| IDC 13-17 years | -0.31 | -0.15 | -0.14 | -0.09 | -0.38 | -0.08 | -0.26 | -0.37 | -0.30 | -0.25 | -0.39 |
| IDC ≥ 18 years | -0.09 | -0.03 | -0.18 | 0.08 | -0.27 | 0.08 | -0.14 | -0.37 | -0.13 | -0.32 | -0.06 |

*Note.* ACA, anterior cerebral artery; MCA, middle cerebral artery; PCA, posterior cerebral artery. Concurrent tyrosine, tryptophan, plasma Phe, and brain Phe were available for 30 participants. IDC = Index of Dietary Control. IDC 0–5 years available for 17 participants, IDC 6–12 years available for 19 participants, IDC 13–17 years available for 21 participants, IDC ≥ 18 years available for 18 participants. None of the correlations reached significance after correction for multiple testing (FDR correction).

**Table S3.** Relationship between CBF and cognition in patients with early-treated PKU (correlation coefficients)

|  | Global CBF | Left MCA | Left MCA anterior | Left  MCA  middle | Left MCA posterior | Left PCA | Right MCA | Right  MCA  anterior | Right  MCA middle | Right MCA posterior | Right PCA |
| --- | --- | --- | --- | --- | --- | --- | --- | --- | --- | --- | --- |
| Processing speed | -0.106 | -0.187 | -0.307 | -0.166 | -0.115 | -0.170 | -0.239 | -0.271 | -0.181 | -0.192 | -0.088 |
| Working memory | -0.075 | -0.093 | -0.144 | -0.026 | -0.133 | -0.125 | -0.041 | -0.082 | -0.060 | 0.015 | 0.062 |
| Inhibition | -0.265 | -0.157 | -0.219 | -0.110 | -0.140 | -0.214 | -0.137 | -0.126 | -0.180 | -0.062 | -0.288 |
| Switching | 0.048 | 0.133 | 0.004 | 0.151 | 0.277 | 0.019 | 0.199 | 0.194 | 0.143 | 0.268 | -0.011 |
| Sustained Attention | 0.118 | 0.190 | 0.113 | 0.216 | 0.220 | 0.168 | 0.229 | 0.261 | 0.161 | 0.145 | -0.075 |
| Alertness | -0.093 | -0.063 | -0.160 | 0.004 | -0.080 | -0.073 | -0.054 | -0.130 | -0.086 | 0.041 | 0.132 |
| Divided attention | -0.058 | 0.011 | 0.166 | -0.046 | -0.073 | -0.020 | -0.048 | 0.044 | -0.067 | -0.126 | -0.160 |

*Note.* ACA, anterior cerebral artery; MCA, middle cerebral artery; PCA, posterior cerebral artery. None of the correlations reached significance after correction for multiple testing (FDR correction).

**Table S4.** Relationship between metabolics, white matter lesion load and cognition in patients with early-treated PKU (correlation coefficients).

|  | | WM lesion score | Phe Plasma | Brain Phe | Tyrosin | Tryptophan |
| --- | --- | --- | --- | --- | --- | --- |
| IQ |  | 0.156 | 0.145 | 0.039 | 0.151 | 0.189 |
| Processing speed |  | 0.140 | 0.182 | 0.072 | 0.281 | 0.054 |
| Working memory |  | 0.295 | -0.019 | 0.072 | 0.001 | 0.035 |
| Inhibition |  | 0.069 | 0.086 | -0.074 | -0.203 | 0.025 |
| Switching |  | 0.089 | -0.134 | -0.284 | -0.007 | -0.042 |
| Sustained attention (SD) |  | 0.237 | 0.044 | 0.091 | 0.103 | 0.337 |
| Sustained attention (M) |  | 0.030 | -0.101 | -0.236 | 0.178 | 0.112 |
| Alertness |  | -0.013 | -0.105 | -0.090 | 0.228 | 0.049 |
| Divided attention |  | 0.081 | 0.191 | 0.105 | 0.074 | -0.013 |
| WM lesion score |  | 1.000 | 0.078 | 0.304 | -0.078 | 0.118 |

*Note.* SD, standard deviation; M, mean; WM, white matter. None of the correlations reached significance.
